# Impact of antibiotic treatment on immune-checkpoint blockade efficacy in advanced non-squamous non-small cell lung cancer

**DOI:** 10.18632/oncotarget.24751

**Published:** 2018-03-27

**Authors:** Florian Huemer, Gabriel Rinnerthaler, Theresa Westphal, Hubert Hackl, Georg Hutarew, Simon Peter Gampenrieder, Lukas Weiss, Richard Greil

**Affiliations:** ^1^ IIIrd Medical Department, Paracelsus Medical University Salzburg, Salzburg, Austria; ^2^ Salzburg Cancer Research Institute (SCRI), Salzburg, Austria; ^3^ Cancer Cluster Salzburg (CCS), Salzburg, Austria; ^4^ Division of Bioinformatics, Biocenter, Medical University of Innsbruck, Innsbruck, Austria; ^5^ Institute of Pathology, Paracelsus Medical University Salzburg, Salzburg, Austria

**Keywords:** immune-checkpoint blockade, antibiotics, non-squamous non-small cell lung cancer, progression-free survival, overall survival

## Abstract

**Introduction:**

Despite durable responses from immune-checkpoint blockade (ICB) in a subset of patients with advanced non-small cell lung cancer (NSCLC), the majority of patients do not derive benefit from this treatment. In this analysis we evaluated the impact of concomitant administration of antibiotics during initiation of ICB on clinical outcome.

**Methods:**

Advanced non-squamous NSCLC patients receiving ICB as second- or later line between 2015 and 2017 at our tertiary cancer center in Salzburg (Austria) were included. Concomitant use of antibiotics was defined as administration of antibiotics within a time frame of one month before or one month after initiation of ICB (AB^+^-group).

**Results:**

Of the 30 patients included, 11 (36.7%) received antibiotics one month before or one month after start of ICB (AB^+^-group). Median PFS on ICB was in favor of the AB^-^-group (AB^-^: 3.1 months [95%CI: 3.0-16.3]; AB^+^: 2.9 months, [95%CI: 1.9-NA]; HR=0.46 [95%CI: 0.12-0.90], p=0.031). Furthermore, median OS was significantly longer in the AB^-^-group (AB^-^: 15.1 months [95%CI: 11.1-NA]; AB^+^: 7.5 months [95%CI: 6.3-NA]; HR=0.31 [95%CI: 0.02-0.78], p=0.026). In a multivariate analysis, the antibiotic treatment status was identified as the only parameter statistically significantly associated with PFS (p=0.028) and OS (p=0.026).

**Conclusions:**

Stratification of patients according to the antibiotic treatment status is warranted in future trials investigating ICB.

## INTRODUCTION

With over 155,000 and 183,000 estimated deaths, lung cancer will be the leading cause of cancer-related mortality in the United States [1] and in Europe [2] in 2017, respectively. In recent years, strategies unleashing pre-existing anti-tumor responses by immune-checkpoint blockade (ICB) have revolutionized cancer therapy and have led to the approval of several immune-checkpoint inhibitors for second-line therapy in advanced non-small cell lung cancer (NSCLC) [3–6]. While programmed death-ligand 1 (PD-L1) expression on tumor cells was predictive of achieving benefit from ICB during second-line treatment of metastatic non-squamous NSCLC, such an association could not been shown in squamous NSCLC [3–5]. Preclinical studies assume an influence of the composition of the gut microbiota on the benefit from ICB by improving dendritic cell function and T-cell activation [7, 8].

In our retrospective monocentric analysis, we tested the influence of antibiotic treatment in temporal proximity to the initiation of ICB on clinical outcome in previously treated advanced non-squamous NSCLC.

## RESULTS

Between May 2015 and November 2017, forty-four patients with advanced non-squamous NSCLC were treated with ICB as second- or later line treatment at our institution and 36 of them were evaluable for treatment outcome based on our inclusion criteria. Overall 6 patients had to be excluded due to ICB treatment within clinical trials with unpublished primary outcomes. Therefore, 30 patients (25 patients treated with nivolumab and 5 patients treated with pembrolizumab) were included in this retrospective analysis.

Baseline characteristics are depicted in Table 1. Eleven patients received antibiotics during start of ICB (AB^+^-group) while the remaining 19 patients did not (AB^-^-group). Application of antibiotics was initiated due to upper respiratory tract infection, fever without a focus or as preoperative antibiotic prophylaxis in 45.5%, 45.5% and 9% of patients. Penicillins (7 of 11, 64%), fluoroquinolones (4 of 11, 36%) and carbapenems (2 of 11, 18%) were the most frequently administered antibiotics. The majority of patients were treated with ICB in second-line (AB^-^ 63.2% *versus* AB^+^ 45.5%, p=0.773). After ICB, subsequent therapy was initiated in 17 patients (58%). The PD-L1 expression status was available in 90% of patients.

**Table 1:** Baseline characteristics of patients with advanced non-squamous NSCLC treated with immune-checkpoint blockade (ICB; n=30)

		AB^+^-group^a^ N (%)	AB^—^group^b^ N (%)	p-value
**Overall**		11 (36.7)^c^	19 (63.3)	
**Sex**	male	7 (63.6)	7 (36.8)	0.257
	female	4 (36.4)	12 (63.2)	
**Anti-PD1 antibody**	nivolumab	8 (72.7)	17 (89.5)	0.327
	pembrolizumab	3 (27.3)	2 (10.5)	
**Activating EGFR mutation**^d^	yes	4 (36.4)	2 (10.5)	0.156
	no	7 (63.6)	16 (84.2)	
	missing	0 (0.0)	1 (5.3)	
**ALK translocation**^d^	yes	1 (9.1)	1 (5.3)	0.476
	no	8 (72.7)	17 (89.4)	
	missing	2 (18.2)	1 (5.3)	
**Prior therapy lines**	1	5 (45.5)	12 (63.2)	0.773
	2	2 (18.2)	4 (21.1)	
	3	1 (9.1)	2 (10.5)	
	4	0 (0.0)	1 (5.3)	
	5	1 (9.1)	0 (0.0)	
**Prior therapy**^e^	platinum-based + pemetrexed	3 (27.3)	10 (52.6)	0.048
	platinum-based + docetaxel or gemcitabine	2 (18.2)	5 (26.3)	
	TKI	5 (45.5)	1 (5.3)	
	other monochemotherapy	0 (0.0)	3 (15.8)	
**Subsequent therapy**	docetaxel	2 (18.2)	4 (21.1)	0.380
	TKI	1 (9.1)	3 (15.7)	
	other chemotherapy/trial	1 (9.1)	6 (31.6)	
	no subsequent therapy	7 (63.6)	6 (31.6)	
**Indication for AB application**	upper respiratory tract infection	5 (45.5)		
	fever without a focus	5 (45.5)		
	Preoperative antibiotic prophylaxis	1 (9.0)		
**PD-L1 status**^f^	Positive	5 (45.5)	10 (52.6)	0.733
	Negative	4 (36.4)	8 (42.1)	
	Missing	2 (18.1)	1 (5.3)	
**PD-L1 status**^f^**(categorical)**	<1%	4 (36.4)	8 (42.1)	0.479
	1-50%	1 (9.1)	5 (26.3)	
	>50%	4 (36.4)	5 (26.3)	
	missing	2 (18.1)	1 (5.3)	

^a^AB^+^-group: patient group that received antibiotics within a time frame of one month before or one month after initiation of immune-checkpoint blockade.

^b^AB^-^-group: antibiotic-naïve patients within a time frame of one month before or one month after initiation of immune-checkpoint blockade.

^c^In one patient transformation of EGFR mutant NSCLC into small-cell lung cancer was histologically proven during subsequent therapy to ICB.

^d^EGFR mutation status and ALK translocation status were routinely assessed. Some patients had missing data on molecular alterations due to consumed tumor material.

^e^Prior therapy in one patient in the AB^+^-group is unknown.

^f^PD-L1 expression status on tumor cells was assessed by immunohistochemistry utilizing the anti-PD-L1 clone 22C3 from Dako^®^. In three patients PD-L1 status could not be assessed due to consumed tumor material.

TKI: tyrosine kinase inhibitor; AB: antibiotics; PD-L1: programmed death-ligand 1; EGFR: epidermal growth factor receptor; ALK: anaplastic lymphoma kinase.

**Figure 1:**
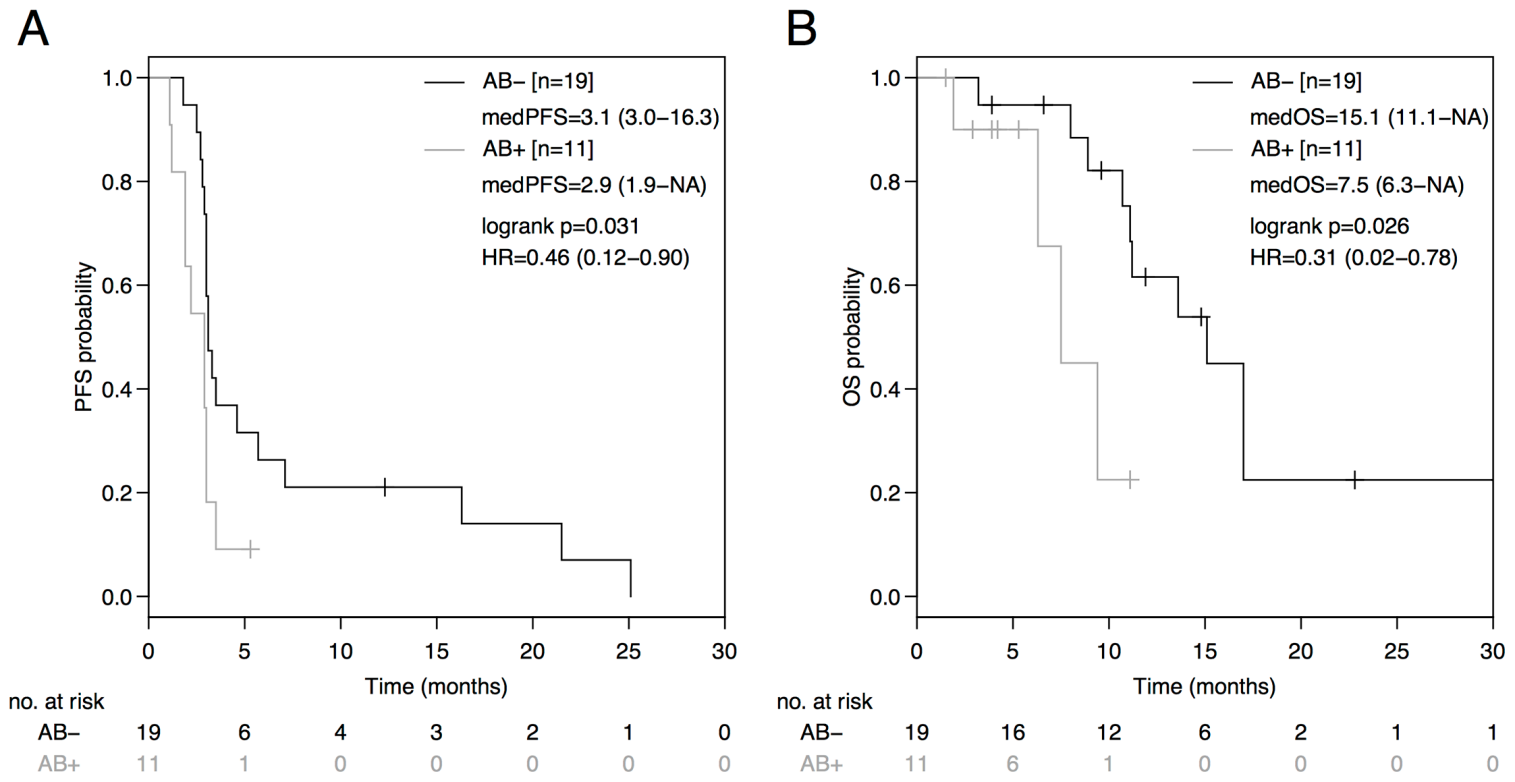
PFS and OS from initiation of immune-checkpoint blockade (ICB) based on antibiotic treatment status Comparison of Kaplan-Meier curves for PFS **(A)** and OS **(B)** between AB^-^ and AB^+^ advanced non-squamous NSCLC groups. HR is hazard ratio, 95% confidence interval in brackets.

Median progression-free survival (PFS) on ICB was statistically significantly in favor of the AB^-^-group (AB^-^: 3.1 months [95%CI: 3.0-16.3]; AB^+^: 2.9 months [95%CI: 1.9-NA]; HR=0.46 [95%CI: 0.12-0.90], p=0.031) with a corresponding 4-month PFS rate of 36.8% *versus* 9.1%, respectively (HR=0.41 [95%CI: 0.11-0.86], p=0.024) as outlined in Figure 1A.

This PFS benefit translated into a superior median overall survival (OS) in the AB^-^-group (AB^-^: 15.1 months [95%CI: 11.1-NA]; AB^+^: 7.5 months [95%CI: 6.3-NA]; HR=0.31 [95%CI: 0.02-0.78] p=0.026; Figure 1B). No patient in the AB^+^-group died due to the infection that had necessitated concomitant antibiotic administration with the initiation of ICB.

**Figure 2:**
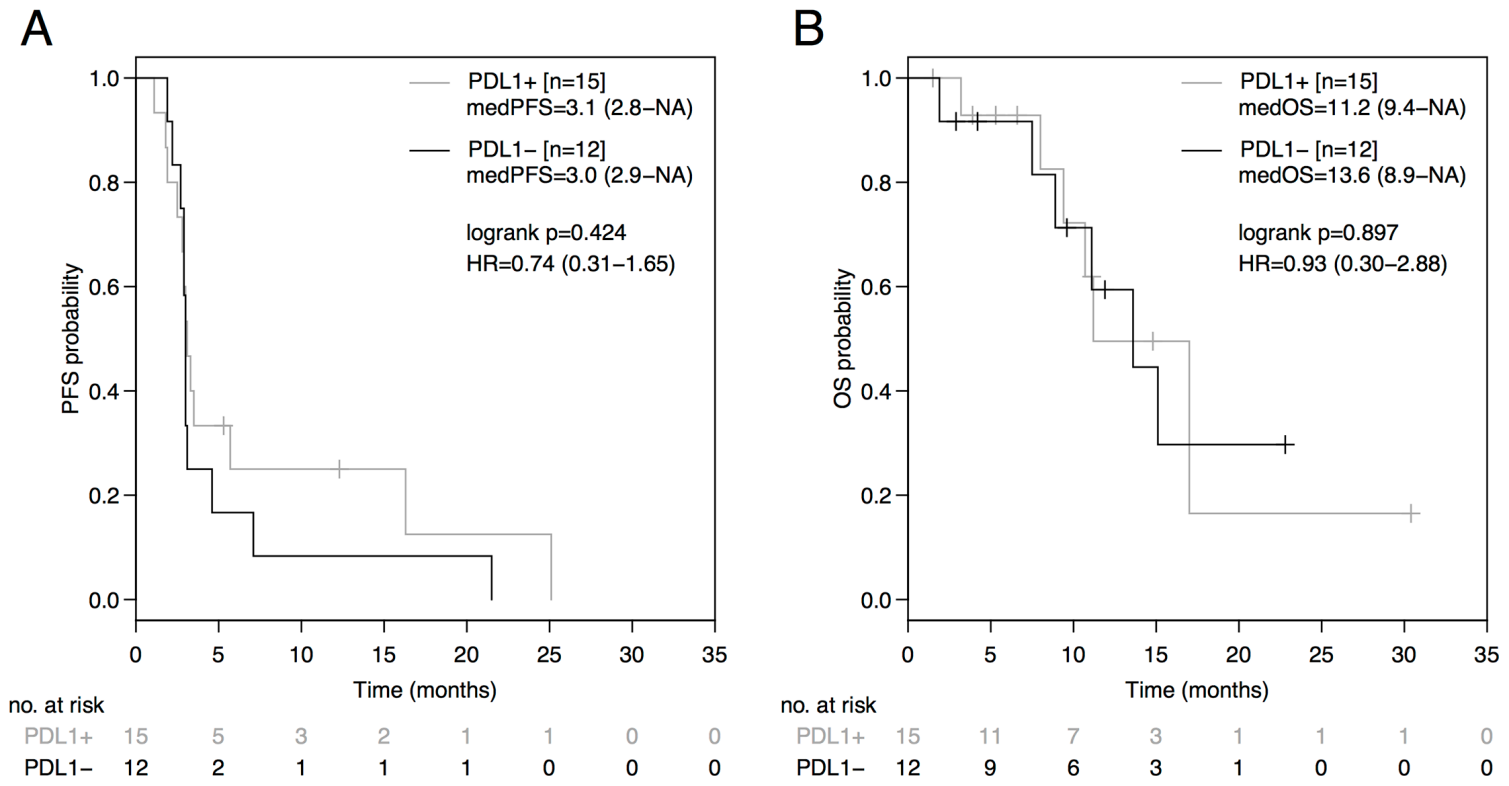
PFS and OS from initiation of immune-checkpoint blockade (ICB) based on the PD-L1 expression status Comparison of Kaplan-Meier curves for PFS **(A)** and OS **(B)** between PD-L1 negative and PD-L1 positive advanced non-squamous NSCLC groups. HR is hazard ratio, 95% confidence interval in brackets.

**Figure 3:**
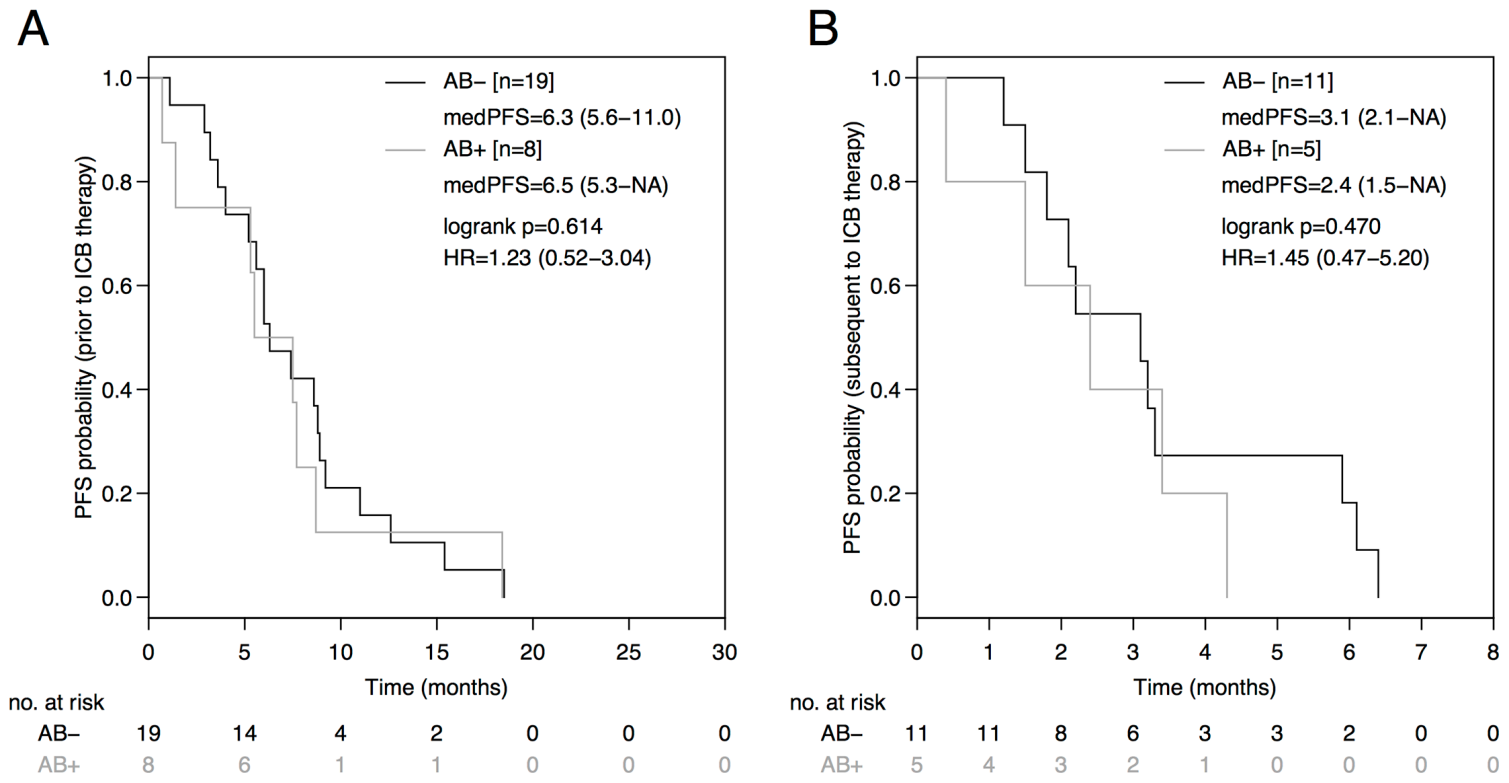
PFS during prior therapy to immune-checkpoint blockade (ICB) and during subsequent therapy based on antibiotic treatment status Comparison of Kaplan-Meier curves for PFS during prior therapy to ICB **(A)** and subsequent therapy **(B)** between AB^-^ and AB^+^ advanced non-squamous NSCLC groups. HR is hazard ratio, 95% confidence interval in brackets.

The PD-L1 status was neither predictive in terms of PFS (PD-L1^+^: 3.1 months [95%CI: 2.8-NA]; PD-L1^-^: 3.0 months [95%CI: 2.9-NA]; HR 0.74 [95%CI: 0.31-1.65], p=0.424; Figure 2A), nor in terms of OS (PD-L1^+^: 11.2 months [95%CI: 9.4-NA]; PD-L1^-^: 13.6 months [95%CI: 8.9-NA]; HR 0.93 [95%CI: 0.30-2.88], p=0.897; Figure 2B) in the entire non-squamous NSCLC cohort. No difference in PFS was observed between the AB^-^ and AB^+^-group during therapy prior to ICB (AB^-^: 6.3 months [95%CI: 5.6-11.0]; AB^+^: 6.5 months [95%CI: 5.3-NA]; HR=1.23 [95%CI: 0.52-3.04], p=0.614; Figure 3A) or during subsequent therapy (AB^-^: 3.1 months [95%CI: 2.1-NA]; AB^+^: 2.4 months [95%CI: 1.5-NA]; HR=1.45 [95%CI: 0.47-5.20], p=0.470; Figure 3B). In a multivariate analysis including the parameters antibiotic treatment status, sex, immune-checkpoint inhibitor, EGFR mutation status, ALK translocation status, number of prior therapy lines, PD-L1 expression status, and immune-related adverse events, the antibiotic treatment status was the only parameter statistically significantly associated with PFS (HR=5.34, p=0.028) and OS (HR=14.81, p=0.026) in our non-squamous NSCLC cohort receiving ICB (Table 2).

**Table 2:** Univariate and multivariate analysis for PFS (A) and OS (B) in patients with advanced non-squamous NSCLC treated with immune-checkpoint blockade

A								
PFS	*univariate*		*multivariate*					
Variable	HR	*P*	levels	n	events	coef	HR	*P*
AB	2.50	**0.032**	yes [n=9] no [n=16]	25	23	1.68	5.34	**0.028**
Sex	1.30	0.50	female [N=13] male [n=12]			0.77	2.17	0.193
ICB	1.42	0.53	pembrolizumab [n=5] nivolumab [n=20]			0.27	1.31	0.691
EGFR mutation	2.42	0.068	yes [n=4] no [n=21]			1.03	2.79	0.131
ALK translocation	0.95	0.95	yes [n=2] no [n=23]			-0.09	0.92	0.921
Number of prior therapy lines	1.04	0.92	≥2 [n=9] ≤ 1 [n=16]			0.57	1.76	0.370
PD-L1 status	1.42	0.39	positive [n=12] negative [n=13]			-0.26	0.77	0.689
IR-AE	0.69	0.35	yes [n=12] no [n=13]			0.95	2.60	0.138

B								
OS	*univariate*		*multivariate*					
variable	HR	*P*	levels	n	events	coef	HR	*P*
AB	4.29	**0.038**	yes [n=9] no [n=16]	25	12	2.70	14.81	**0.026**
Sex	1.300	0.64	female [N=13] male [n=12]			2.04	7.67	0.079
ICB	<0.1	1.00	pembrolizumab [n=5] nivolumab [n=20]			-20.0	<0.1	0.999
EGFR mutation	2.59	0.12	yes [n=4] no [n=21]			0.22	1.25	0.786
ALK translocation	2.02	0.38	yes [n=2] no [n=23]			0.90	2.46	0.374
Number of prior therapy lines	1.23	0.71	≥2 [n=9] ≤1 [n=16]			-0,62	0.54	0.462
PD-L1 status	1.05	0.94	positive [n=12] negative [n=13]			-0,59	0.55	0.470
IR-AE	0.65	0.43	yes [n=12] no [n=13]			1.27	3.56	0.139

AB: administration of antibiotics within a time frame of one month before to one month after start of immune checkpoint-blockade; ICB: immune checkpoint blocker; EGFR: epidermal growth factor receptor; ALK: anaplastic lymphoma kinase; PD-L1: programmed death-ligand 1; IR-AE: immune-related adverse events; coeff is the coefficient of the corresponding variable in the Cox regression model, HR is hazard ratio.

During treatment with immune-checkpoint inhibitors immune-related adverse events occurred in 57.9% and 27.3% in AB^-^ and AB^+^ patients, respectively. Only grade ≤ 2 adverse events were documented whereas three patients (15.8%) in the AB^-^ group necessitated systemic cortisone administration resulting in a temporary ICB pause in two of these patients. One patient had to discontinue ICB due to a prolonged therapy-induced pneumonitis (Table 3).

**Table 3:** Immune-related adverse events in patients with advanced non-squamous NSCLC treated with immune-checkpoint blockade (ICB; n=30)

		AB^+^-group^a^ n=11 (36.7%)	AB^—^group^b^ n=19 (63.3%)	p-value
ICB toxicity^*^ **grade ≥ 1**	Yes	3 (27.3)	11 (57.9)	0.466
	No	8 (72.7)	8 (42.1)	
ICB toxicity^*^ **grade**	1	2 (18.2)	8 (42.1)	0.142
	2	1 (9.1)	3 (15.8)	
	≥3	0 (0.0)	0 (0.0)	
ICB organ related toxicity^*^	Hepatitis	1 (9.1)	2 (10.5)	1.000
	Colitis	0 (0.0)	0 (0.0)	
	Pneumonitis	0 (0.0)	2 (10.5)	
	Thyreoiditis	2 (18.2)	4 (21.1)	
	Skin/itch	0 (0.0)	3 (15.8)	
	Hypophysitis	0 (0.0)	0 (0.0)	
**Toxicity related measures**	ICB delay	0 (0.0)	3 (15.8)	
	ICB stop	0 (0.0)	1 (5.3)	
	Systemic cortisone use	0 (0.0)	3 (15.8)	

ICB: Immune-checkpoint blockade; toxicity grade based on the Common Terminology Criteria for Adverse Events version 4.03.

## DISCUSSION

According to a large epidemiologic study comparing 125,441 cases and 490,510 controls recurrent antibiotic exposure and associated bacterial dysbiosis promotes cancer formation in various organs [9]. The risk of developing lung cancer was increased by 1.4-fold when more than 5 courses of penicillin were administered. Animal studies suggest that the relative abundance of certain bacterial species such as *Bifidobacterium* is crucial for dendritic cell maturation, enhanced cytotoxic T-lymphocyte (CTL) priming, accumulation of CTL in the tumor microenvironment and finally for the efficacy of ICB [8]. Due to immunosuppressive effects of the underlying malignancy itself and chemotherapy induced temporary leukopenia, cancer patients are prone to bacterial infections and often necessitate antibiotic treatment [10]. Our analysis of an unselected, consecutive advanced non-squamous NSCLC cohort demonstrated a statistically significant and clinically meaningful PFS (HR 0.46; p=0.031) and OS (HR 0.31; p=0.026) advantage for the AB^-^-group during initiation of ICB when compared to the AB^+^-group. In order to rule out differences in biologic aggressiveness between the AB^-^ and AB^+^-group, PFS during prior therapy (HR 1.23; p=0.614) and during subsequent therapy (HR=1.45; p=0.470) were compared without detecting a statistically significant difference between the groups (Figure 3). It is noteworthy, that more patients in the AB^-^-group (68%) received subsequent therapy after ICB in comparison to the AB^+^-group (36%), which might have affected OS results. Non-squamous NSCLC patients harboring an activating mutation of the epidermal growth factor receptor (EGFR) gene might derive less benefit from ICB as previously reported [4, 11]. However, according to the multivariate analysis only the antibiotic treatment status had a statistically significant impact on PFS and OS, while this was not the case for several other parameters such as EGFR mutation or PD-L1 expression status (Table 2).

Clinical data on the influence of antibiotics on ICB efficacy are sparse. Routy et al. have recently reported on the influence of antibiotic use on ICB efficacy including various types of cancer [12]. In a subset analysis, they described a PFS and OS disadvantage for the NSCLC cohort receiving antibiotics two months before or within one month after ICB initiation (median PFS: AB^+^: 3.5 months versus AB^-^ 4.1 months, p=0.017; median OS: AB^+^ 11.5 months *versus* AB^-^ 20.6 months, p<0.001) [12]. Furthermore, a relative abundance of *Akkermansia muciniphila* in the stool of patients was associated with clinical response in NSCLC patients receiving ICB. Fecal microbiota transplantation of responders stools into antibiotics-pretreated mice conferred sensitivity whereas stools from non-responders conveyed resistance to PD-1 blockade. In a translational approach, Type 1 T helper cell and cytotoxic T lymphocyte cell reactivity against *Akkermansia muciniphila* and increased interferon-gamma levels correlated with clinical outcome.

In conclusion, our study demonstrated that a PFS and OS benefit derived from ICB may be attenuated by the administration of antibiotics in temporal proximity to initiation of ICB in advanced non-squamous NSCLC. Our observations are of major importance in a field, where very expensive but effective drugs such as immune-checkpoint inhibitors are still seeking adequate biomarkers and optimized guidelines for their application. Despite the preliminary evidence of these findings, strict indications for the use of antibiotics in temporal proximity to ICB initiation should be recommended. Furthermore, stratification according to antibiotic treatment status may be warranted in future trials investigating ICB.

## MATERIALS AND METHODS

Patients with histologically confirmed advanced non-squamous NSCLC treated with ICB as second- or later line between 2015 and 2017 at our tertiary cancer center in Salzburg, Austria, were included in this retrospective analysis. Patients were defined as evaluable if at least one radiologic reassessment after treatment initiation was available. Radiologic reassessment by PET-CT or CT scan was performed every 2 to 3 months, or as clinically indicated. Immune-related adverse events were assessed based on the Common Terminology Criteria for Adverse Events (CTCAE) version 4.03. PFS was calculated from the date of start of ICB until radiologically confirmed progression or death. OS was calculated from the date of start of ICB until date of death or date of last known follow-up. Concomitant (also termed as “in temporal proximity”) use of antibiotics was defined as application of antibiotics within a time frame of one month before or one month after initiation of ICB (AB^+^-group) opposed to antibiotic-naïve patients within the same time frame (AB^-^-group). PD-L1 expression status on tumor cells was assessed by immunohistochemistry utilizing the anti-PD-L1 clone 22C3 from Dako®. PD-L1 positivity was defined as PD-L1 expression in ≥1% of tumor cells. Differences in patient baseline characteristics between the AB^+^-and AB^-^-group were tested by two-sided Fisher’s exact test. Survival curves were estimated by the Kaplan–Meier method. Log-rank test was used to compare survival distributions between two patient groups. Cox regression models were used for univariate and multivariate analyses of PFS and OS including the parameters antibiotic treatment status, sex, immune-checkpoint inhibitor, EGFR mutation status, ALK translocation status, number of prior therapy lines, PD-L1 expression status, and immune-related adverse events. Proportional hazard assumptions were tested. All analyses were performed using the statistical software environment R (version 3.4.1) including package ‘survival’.

## Abbreviations

CTL: cytotoxic T-lymphocyte
EGFR: epidermal growth factor receptor
ICB: immune-checkpoint blockade
NSCLC: non-small cell lung cancer
OS: overall survival
PD-L1: programmed death-ligand 1
PFS: progression-free survival
